# Priming of Immune System in Tomato by Treatment with Low Concentration of L-Methionine

**DOI:** 10.3390/ijms25126315

**Published:** 2024-06-07

**Authors:** Tomoya Tanaka, Moeka Fujita, Miyuki Kusajima, Futo Narita, Tadao Asami, Akiko Maruyama-Nakashita, Masami Nakajima, Hideo Nakashita

**Affiliations:** 1 Graduate School of Bioscience and Biotechnology, Fukui Prefectural University, Fukui 910-1195, Japan; s2273014@g.fpu.ac.jp (T.T.); mfujita@agr.kyushu-u.ac.jp (M.F.);; 2 Graduate School of Bioresource and Bioenvironmental Sciences, Kyushu University, Fukuoka 819-0395, Japan; amaru@agr.kyushu-u.ac.jp; 3 Graduate School of Agricultural and Life Sciences, The University of Tokyo, Tokyo 113-8567, Japan; kusajima@g.ecc.u-tokyo.ac.jp (M.K.); asami@g.ecc.u-tokyo.ac.jp (T.A.); 4 Graduate School of Agriculture, Ibaraki University, Ibaraki 300-0393, Japan; masami.nakajima.pp@vc.ibaraki.ac.jp

**Keywords:** methionine, phytohormone, priming, disease resistance, tomato

## Abstract

Various metabolites, including phytohormones, phytoalexins, and amino acids, take part in the plant immune system. Herein, we analyzed the effects of L-methionine (Met), a sulfur-containing amino acid, on the plant immune system in tomato. Treatment with low concentrations of Met enhanced the resistance of tomato to a broad range of diseases caused by the hemi-biotrophic bacterial pathogen *Pseudomonas syringae* pv. *tomato (Pst*) and the necrotrophic fungal pathogen *Botrytis cinerea* (*Bc*), although it did not induce the production of any antimicrobial substances against these pathogens in tomato leaf tissues. Analyses of gene expression and phytohormone accumulation indicated that Met treatment alone did not activate the defense signals mediated by salicylic acid, jasmonic acid, and ethylene. However, the salicylic acid-responsive defense gene and the jasmonic acid-responsive gene were induced more rapidly in Met-treated plants after infection with *Pst* and *Bc*, respectively. These findings suggest that low concentrations of Met have a priming effect on the phytohormone-mediated immune system in tomato.

## 1. Introduction

Systemically induced disease resistance in plants is an important self-defense mechanism against pathogens, which are activated by various types of stimuli and protect the plant body from a broad range of attackers. Some of these resistance mechanisms activated by pathogens are induced through phytohormone-governed signaling pathways. The salicylic acid (SA)-mediated defense response and jasmonic acid (JA)-mediated defense response are effective against biotrophic and necrotrophic pathogens, respectively [1,2]. Systemic acquired resistance (SAR) is induced through the SA-mediated signaling pathway after pathogen infection and is accompanied by the expression of defense-related genes, such as pathogenesis-related (PR) genes, even in healthy leaves [3,4,5,6,7]. Since SAR is a strong defense system against subsequent pathogenic attacks, it has been used in the field by exploiting plant activators that induce SAR [8,9,10,11]. Among plant activators, probenazole (PBZ, 3-allyloxy-1,2-benzisothiazole-1,1-dioxide) and its derivative 1,2-benzisothiazol-3(2*H*)-one1,1-dioxide (BIT) are capable of activating the SAR signaling pathway upstream of SA biosynthesis [8,12,13]. On the other hand, tiadinil (TDL, 5-(3-chloro-4-methylanilinocarbonyl)-4-methyl-1,2,3-thiadiazole) and benzo(1,2,3)thiadiazole-7-carbothioic acid *S*-methyl ester (BTH) act downstream of SA biosynthesis in the SAR signaling pathway [9,10,14]. Despite the protective effect against pathogens, SA-mediated defense signaling for SAR induction reduces plant growth [15]. Similarly, JA is also a phytohormone that inhibit plant growth, especially root growth [16,17].

Unlike previously mentioned resistance mechanisms, which have a trade-off relationship with growth, the priming mechanism of the plant immune system is attracting increasing attention because it does not inhibit plant growth and has a protective effect against pathogens [18]. Priming was previously reported as a type of resistance mechanism in plants interacting with symbiotic or nonpathogenic microorganisms, such as the arbuscular mycorrhizal fungi *Rhizophagus irregularis* [19,20], *Funneliformis mosseae* [21,22], and *Gigaspore margarita* [23,24]; the endophytic bacterium *Azospirillum* sp. B510 [25]; and the non-pathogenic rhizobacteria *Pseudomonas fluorescens* WCS417r [26,27], *Bradyrhizobium* sp. ORS278 [28], and *Pseudomonas aeruginosa* 7NSK2 [29]. While primed plants can respond more rapidly and strongly to pathogen infection to protect themselves, the expression of the major defense-related genes induced by SA- or JA-mediated signaling is absent or very low before pathogen infection. Priming has been also reported to be activated by treatment with chemicals, such as a synthetic strigolactone GR24 [30], *β*-aminobutyric acid (BABA) [31], and (*R*)-β-homoserine (RBH) [32], although BABA represses plant growth. 

Many reports have described the important roles of proteinogenic amino acids in the plant immune system. The metabolism and transport of L-glutamine plays a crucial role in disease resistance in *Arabidopsis* [33]. In rice, treatment of roots with L-glutamic acid induces disease resistance to rice blast caused by *Magnaporthe oryzae* [34]. Treatment of tomato and *Arabidopsis* roots with L-histidine induces resistance to bacterial wilt caused by *Ralstonia solanacearum* [35]. 

Since L-methionine (Met), a sulfur-containing amino acid, is a precursor of the phytohormone ethylene (ET) that plays important roles in disease resistance and development, a lot of efforts have been made to clarify the functions of Met in the plant immune system. In pearl millet, exogenous application of 10 or 15 mM Met induces resistance to downy mildew caused by *Sclerospora graminicola* [36]. In grapevine, foliar treatment with 30 mM Met reduces infection by *Plasmopara viticola* [37]. Treatment of the first true leaves of tomato plants with a lower concentration of Met (0.67 mM) induces resistance to powdery mildew in the upper leaves and *Fusarium* wilt diseases in roots [38,39]. On the other hand, Met concentrations above 5 mM have been reported to inhibit spore germination and the mycelial growth of the necrotrophic fungus *Botrytis cinerea* (*Bc*) [40]. Therefore, the mechanism of disease resistance induced by the lower concentration of Met is of interest. In this study, we evaluated the effects of spray-treatment of all leaves of tomato plants with low concentrations of Met on resistance to foliar diseases: bacterial speck caused by the hemi-biotrophic bacterial pathogen *Pseudomonas syringae* pv. *tomato* DC3000 (*Pst*) [25,28] and gray mold caused by *Bc* [24]. To determine the physiological changes in the leaves of Met-treated tomato plants, the expression of defense-related genes and accumulation of phytohormones were analyzed. Furthermore, the defense responses upon pathogen inoculation in Met-treated plants were investigated by time course analyses of gene expression. 

## 2. Results

### 2.1. Induction of Resistance to a Broad Range of Diseases in Tomato by Low Concentrations of L-methionine

**Figure 1:**
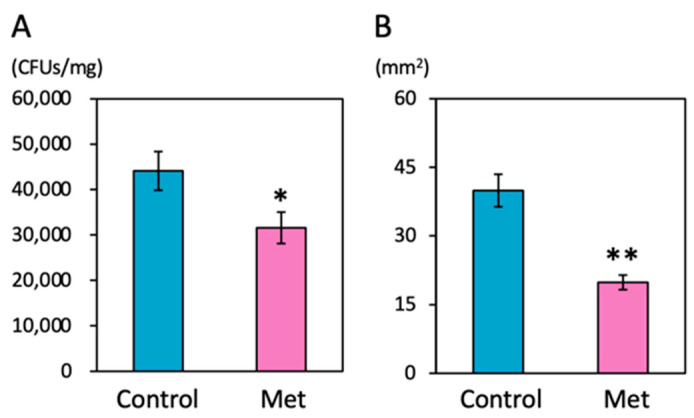
Induction of resistance to tomato leaf speck disease and gray mold disease by L-methionine treatment. (**A**) The growth of *Pst* in tomato leaf tissues. CFUs, colony forming units. Values are shown as the means (±SEs) (*n* = 10). The asterisk indicates a statistically significant difference between data from the control and Met-treated plants (*, *p* < 0.05). (**B**) The area of spreading lesions caused by *Bc*. Values are shown as the means (±SEs) (*n* = 25). Asterisks indicate statistically significant differences between data from the control and Met-treated plants (**, *p* < 0.01).

Resistance to *Pst* was determined by measuring bacterial growth in leaf tissues at two days post challenge inoculation. Treatment with 0.67 mM Met by foliar spaying significantly inhibited bacterial growth in the infected tissues relative to the water-treated control plants (about a 30% reduction from the control) (Figure 1A). No antibacterial activity against *Pst* was detected in leaf extracts of Met-treated plants (Figure S1A). 

The effect of Met treatment on *Bc* infection was estimated by the size of necrotrophic lesions that appeared on the infected leaves three days after drop inoculation. Foliar treatment with 0.67 mM Met two days prior to challenge inoculation significantly reduced the lesion area compared to the water-treated control plants (about a 50% reduction from the control) (Figure 1B). The leaf extracts of Met-treated plants did not exhibit any antifungal activity against *Bc* (Figure S1B). 

### 2.2. Defense-Related Signaling in the L-Methionine-Treated Tomato Plants 

**Figure 2:**
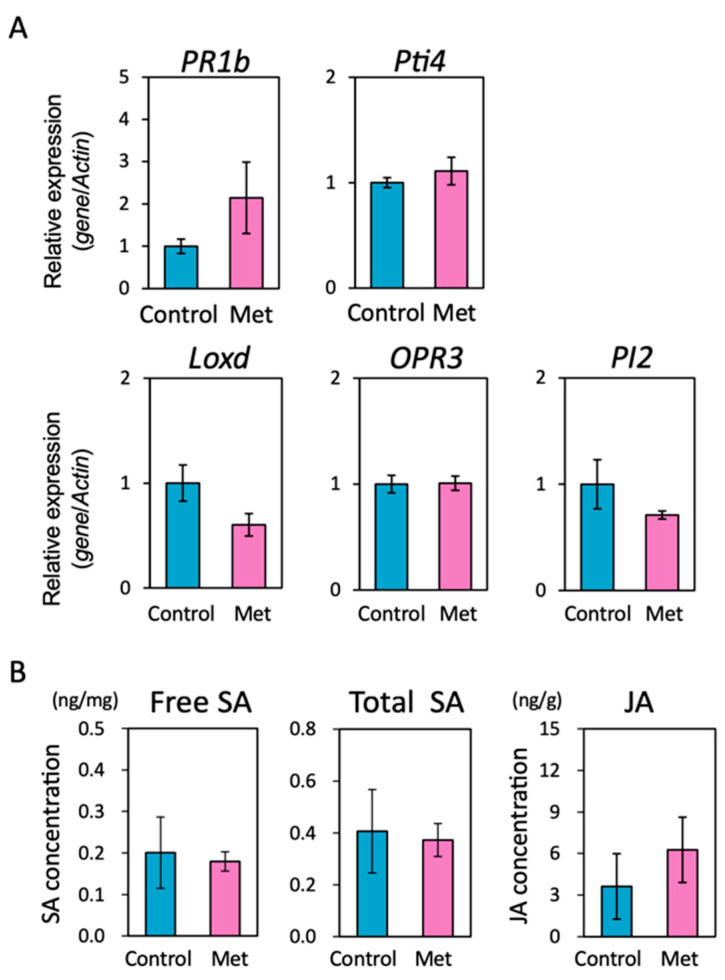
Effects of a low concentration of L-methionine on defense gene expression and phytohormone levels in tomato plants. (**A**) Expression of defense-related genes in L-methionine-treated tomato plants. Values presented are the means (±SEs) (*n* = 8). No significant differences at the *p* < 0.05 level were detected in gene expression between the control and Met-treated plants. (**B**) Phytohormone levels in L-methionine-treated tomato plants. Values presented are the means (±SEs) (*n* = 4). No significant differences at the *p* < 0.05 level were detected in phytohormone levels between the control and Met-treated plants.

The expression of the SA-responsive gene *PR1b* was not influenced by treatment with Met (Figure 2A). The levels of free SA and total SA (free SA + SA-glucoside) in Met-treated plants were not significantly different from those in the water-treated control plants (Figure 2B). Thus, treatment with the low concentration of Met did not activate SA-mediated defense signaling in tomato plants.

JA-mediated defense signaling takes part in resistance to necrotrophic pathogens and the induction of another type of systemically induced disease resistance. Treatment with the low concentration of Met had no effect on the expression of the JA biosynthesis genes *LOXd* (encoding lipoxygenase) and *OPR3* (encoding 12-oxophytodienoate reductase 3), or the JA-responsive gene *PI2* (encoding a protease inhibitor 2) (Figure 2A). Endogenous JA levels were similar between Met-treated plants and the water-treated control plants (Figure 2B). Thus, JA-mediated signaling was not activated by treatment with the low concentration of Met. 

Next, we examined the effect of Met treatment on ET-mediated signaling, because ET is synthesized from Met and plays an important role in disease resistance. Treatment with Met had no effect on the expression of the ET-responsive gene *Pti4* (encoding ethylene responsive factor) (Figure 2A), indicating that ET-mediated signaling was not activated by the low concentration of Met. 

### 2.3. Accelerated Responses to Pathogen Infections in the L-Methionine-Treated Tomato Plants

**Figure 3:**
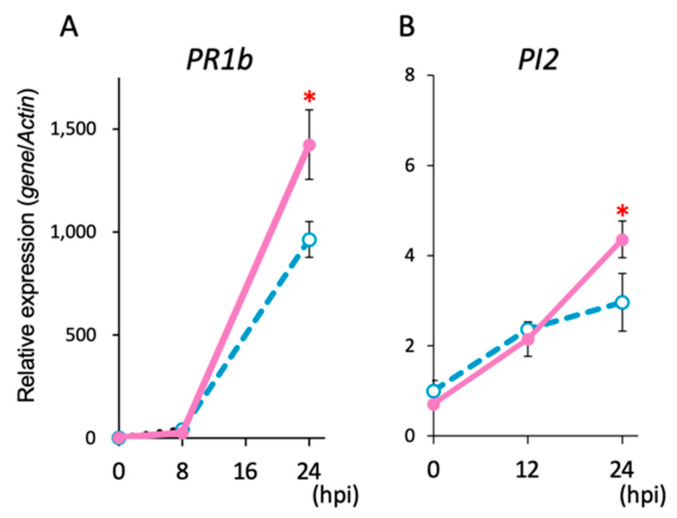
Expression of defense-related genes after infection with pathogens. (**A**) Expression of the *PR1b* gene after infection with *Pst*. (**B**) Expression of the *PI2* gene after infection with the *Bc*. Values presented are the means (±SEs) (*n* = 8). Open circles, water-treated control plants; closed circles, Met-treated plants. Asterisks indicate statistically significant differences between data from the water-treated and Met-treated plants at each time point (*, *p* < 0.05).

After *Pst* inoculation, the expression levels of the SA-related gene *PR1b*, a marker gene for the SA signal-dependent resistance, increased from 8 h post infection (hpi) in both Met-treated and water-treated control plants, but, at 24 hpi the expression level of *PR1b* in Met-treated plants was approximately 50% higher than that in water-treated control plants (Figure 3A). This result indicated that activation of the SA-mediated signaling pathway in response to *Pst* infection was accelerated in Met-treated plants compared to that in the water-treated control plants. Activation of JA-mediated signaling was examined after inoculation with the necrotrophic fungal pathogen *Bc*. Expression levels of the JA-related gene *PI2*, a marker gene for the JA signal-dependent resistance, gradually increased after *Bc* infection in both Met-treated and water-treated control plants. These expression levels were similar at 8 hpi, but at 24 hpi, the level in Met-treated plants was about 50% higher than in water-treated control plants (Figure 3B). This indicated that activation of the JA-mediated signaling pathway in response to *Bc* infection was enhanced in Met-treated plants compared to that in the water-treated control plants.

## 3. Discussion

This study demonstrated that treatment with a low concentration of Met primed the immune system of tomato plants. The tomato plants primed by Met exhibited enhanced resistance against both the hemi-biotrophic bacterial pathogen *Pst* and the necrotrophic fungal pathogen *Bc*, without the production of any antimicrobial substances against these pathogens. The expression of defense-related genes and phytohormone accumulation indicated that Met treatment alone did not activate the defense signals mediated by SA, JA, or ET, suggesting that the low concentration of Met induced a resistance mechanism different from known systemically induced disease resistance mechanisms. However, activation of SA-mediated defense signaling by infection with *Pst* and JA-mediated defense signaling by *Bc* was accelerated in the Met-treated plants compared to the control plants, suggesting that a rapid and strong response to pathogens is responsible for the enhanced disease resistance in the primed plants.

As previously reported, tomatoes primed by mycorrhizal symbiosis with *G. margarita* showed disease resistance to *Pst* and *Bc* and promoted the activation of SA- and JA-mediated signaling upon infection with pathogens [24]. This suggest that a similar priming mechanism is induced by Met and mycorrhizal symbiosis. Since mycorrhizal symbiosis induces disease resistance in distal leaves, it is possible that Met functions in the priming mechanism by mycorrhizal symbiosis, but this remains to be elucidated. Not only SA-mediated defense signaling against infection with compatible *Pst* but also the activation of JA-mediated defense signaling by infection with incompatible *Pseudomonas syringae* pv. *oryzae* was promoted in tomato primed by mycorrhizal colonization [24].

In nature, small numbers of cells of incompatible pathogens should frequently enter plant tissues, but after invasion, when they proliferate to about the 1 × 10^6^ cells/mL level, a hypersensitive response (HR), including plant cell death, is induced as a defense response against these pathogens. Thus, the fact that priming is effective even against incompatible pathogens is presumed to be one of survival strategies in nature to protect against invasion by various microorganisms, thereby avoiding leaf damage by suppressing the growth of incompatible bacteria in plant tissues to the levels that cause HR. Therefore, it would be very interesting to see if Met functions in this survival strategy.

In the ET biosynthetic pathway in plant cells, *S*-adenosyl methionine (SAM) produced from Met is converted to 1-aminocyclopropane-1-carboxylic acid (ACC) and then to ET. In tomato, foliar treatment with the ET biosynthetic precursors SAM (0.2 mM) and ACC (0.1 mM) has been reported to induce disease resistance against *Bc* [41], and treatment with ACC (0.1 or 1 mM) induces ET biosynthesis [42], suggesting that Met induces resistance through ET production. On the other hand, several reports describe that the Met cycle plays important roles in disease resistance to viruses [43,44,45] and bacterial pathogens [46,47].

Met cycle is an important metabolic pathway that receives methyl groups from folate metabolism and uses them for the methylation of DNA and histones, which is also important for ET synthesis in plants [48]. In this cycle, Met reacts with adenosine triphosphate (ATP) to produce SAM, which is a general methyl donor for the methylation of various molecules and a precursor of ET in plants. By transferring a methyl group to the target molecule, SAM is converted to *S*-adenosyl homocysteine (SAH). SAH is converted to homocysteine, which is then supplied with a methyl group from 5-methyltetrahydrofolate to produce Met. The importance of *S*-adenosylhomocysteine hydrolase (SAHH), an enzyme catalyzing the hydrolysis reaction from SAH to adenosine and homocysteine in the Met cycle, in the plant immune system has been reported [47]. While SAHH1 expression is reduced after infection with *Pst*, SAHH2 and SAHH3 are induced after infection with *Bc*. Silencing of all three SAHHs in tomato results in enhanced resistance to *Pst* and the activation of the SA-related defense genes *PR1b*, *PR-P2,* and *PR5*, which does not alter the resistance to *Bc* [47]. Thus, SAHHs have a positive effect on resistance to the necrotrophic pathogen *Bc* and conversely a negative effect on resistance to the hemi-biotrophic pathogen *Pst*. Because homocysteine is a Met precursor in the Met cycle, these different effects on necrotrophs and hemi-biotrophs are presumably due to ET-mediated signaling activated by Met production. On the other hand, the priming by treatment with a low concentration of Met shown here had positive effects on both *Pst* and *Bc*. Therefore, the priming by Met in tomato should involve the activation of a different immune mechanism from that activated by the Met cycle.

In rice, it has been reported that root-dip treatment with 1 to 25 mM of Met for 48 h induces resistance to rice blast disease caused by the hemi-biotrophic fungal pathogen *Magnaporthe oryzae* and that this effect of Met is suppressed by pretreatment with 2-aminoethoxyvinyl glycine (AVG), an ET biosynthesis inhibitor [49,50]. In addition, the production of ET is induced by the Met treatment in rice. In contrast, the resistance in tomato shown here was enhanced by a much lower concentration of Met and without the activation of ET-mediated signaling. Thus, the priming by Met in tomato shown here is likely due to a different mechanism from the reported Met-induced disease resistance in rice.

In *Arabidopsis*, it has been reported that resistance to *Pst* is enhanced by the suppression of folate metabolism that produces the substrate for Met synthase (METS1) and by the suppression of METS1 activity, which are SA-independent disease susceptibility/resistance mechanism [51]. It remains to be clarified whether this mechanism is related to the resistance to *Pst* in the tomatoes primed by Met shown here.

The data presented in this paper demonstrated that a low concentration of Met induced a novel type of Met-induced resistance that was due to the priming of the immune system by activating unknown mechanisms. Gene expression and phytohormone analyses have shown that treatment with a low concentration of Met alone does not activate defense-related signals under our experimental conditions (Figure 2), and a similar phenomenon was observed in the priming of SA-mediated defense mechanism by strigolactone (SL)-mediated signaling [30]. The mechanisms of these priming effects may be similar, although it has not been determined whether the SL-mediated priming is effective against necrotrophic pathogens. The molecular mechanism of enhanced activation of defense response signals mediated by SA and JA after pathogen infection in these primed plants has not been clarified, which is of great importance both scientifically and for the development of plant protection technology. After confirming the effect of Met on *Arabidopsis*, an analysis using various *Arabidopsis* mutants would be useful to elucidate the mechanism of the defense response enhancement.

## 4. Materials and Methods

### 4.1. Plant Growth Conditions and Treatment

Tomatoes (*Solanum lycopersicum* L. cv. Momotaro, Takii & Co., Ltd., Kyoto, Japan) were grown in sterilized potting soil in plastic pots (6 cm × 6 cm × 6 cm) in a growth chamber (16:8 h L/D, 120 μmol/m^2^ s, 25 °C, 60% RH). Cultures were conducted simultaneously under the same conditions with sets of 20 or 48 plants in a single chamber, from which plants of the same growth size were selected for each experiment. Sets of plants were prepared specifically for each experiment and used for only one analysis. All leaves of three-week-old tomato plants were treated with 0.67 mM Met (3 mL/plant) by foliar spraying and incubated under the same condition until analysis. Control plants were pretreated with water. The 3rd compound leaf from the bottom of each plant was used for analyses.

### 4.2. Pathogen Inoculation Assays

For the pathogen inoculation assays, two sets of 5 tomato plants of equal size were selected from 20 cultivated plants and treated with Met or water, respectively.

A challenge inoculation with *Pst* was performed two days after treatment with Met or water. A bacterial suspension (1 × 10^3^ CFUs/mL) of *Pst* was prepared using 10 mM MgCl_2_ after culture in nutrient broth (Eiken Chemical, Tokyo, Japan) containing rifampicin (100 μg/mL) at 30 °C for 24 h. Infiltration with *Pst* was performed using a 1 mL syringe without a needle into an area of at least 2 cm^2^ in the terminal and adjacent leaflets of the 3rd compound leaves. Leaf disks (4 mm in diameter) were taken from the infiltrated part of the leaflet two days after inoculation. Bacterial cells were extracted by homogenizing the leaf disks in 10 mM MgCl_2_ (5 disks from a single plant per sample, 2 samples from each plant, 10 samples in total). Colony forming units (cfus) were estimated by culturing bacterial cells in nutrient broth agar plates after dilution. The mean of 10 samples from Met-treated plants was compared to that of the control. 

A challenge inoculation with *Bc* was performed with the 3rd compound leaves cut from tomato plants two days after chemical treatment. *Bc* strain TV335 [25] was grown and maintained on a potato dextrose agar (Becton, Dickinson and Company, Franklin Lakes, NJ, USA) plate at 20 °C. Spore formation was performed by illuminating the culture plate with near-ultraviolet light (FL15B, Toshiba Lighting & Technology Co., Tokyo, Japan) for three days. Spores were collected by washing the surface with sterilized distilled water. Drop inoculation was performed by placing 5 μL of the spore suspension (1 × 10^6^ spores/mL in 1.2% potato dextrose broth) on the surface of all leaflets of the excised 3rd compound leaves (normally 5 leaflets each). The infected leaves were incubated at 22 °C for two days in the dark with 100% humidity. The size of lesions that appeared on the infected leaflets was measured using ImageJ 1.53t software (National Institute of Health, Bethesda, MD, USA). The average size of the lesions was compared between Met-treated and water-treated control plants (25 lesions in 5 plants). 

### 4.3. Analysis of Phytohormone Levels

For analyses of SA or JA levels, two sets of 4 tomato plants of equal size were selected from 20 cultivated plants and treated with Met or water, respectively. Plant tissue (approximately 100 mg) was collected from the terminal and adjacent leaflets of the 3rd compound leaf into a plastic tube 2 days after treatment (one sample from each plant). Plant tissues were immediately frozen and stored at −80 °C until analysis.

For the analysis of free and total SA (free SA + SA-glucoside) levels, plant tissues (approximately 100 mg) were powdered in liquid nitrogen and used for extraction and analysis. The extraction and measurement of SA levels were performed as previously described [30]. The experiment was repeated two times.

For the analysis of JA levels, plant tissues (approximately 100 mg) were powdered in liquid nitrogen and used for extraction. After adding deuterium-labeled JA (20 ng) to each sample, samples were extracted with 700 μL of 80% acetonitrile and 5% formic acid (FA) (*v*/*v*) and then twice with 500 μL of the same solvent. These extracts were combined and dried in vacuo, which were used for JA purification using solid-phase extraction columns as previously reported [52]. The purified samples were dissolved in 50 μL of 10% methanol in water (*v*/*v*) containing 10 mM FA, and 15 μL of each sample was injected onto a reverse-phase ACQUITY UPLC HSS C18 column (2.1 mm × 100 mm, 1.8 μm; Waters, Milford, MA, USA) in the LC-MS/MS system. The LC-MS/MS analysis and peak annotation were performed as previously reported [52]. The experiment was repeated two times.

### 4.4. Gene Expression Analysis

For the gene expression analysis in the leaves of the Met-treated and control plants, two sets of 4 tomato plants of equal size were selected from 20 cultivated plants and treated with Met or water, respectively. Leaf tissues were taken from terminal and adjacent leaflets of the 3rd compound leaf 2 days after treatment and used for the gene expression analysis (2 samples from a single plant). These were powdered in liquid nitrogen and used for total RNA extraction using Sepasol-RNA I super reagent (Nacalai Tesque, Kyoto, Japan), followed by cDNA synthesis using the PrimeScript RT reagent Kit with gDNA Eraser (Takara Bio, Shiga, Japan). Quantitative RT-PCR using gene-specific primers and cDNA as a template was performed using a LightCycler 96 System (Roche, Basel, Switzerland). PCR conditions were 40 cycles of 5 s at 95 °C and 20 s at 60 °C. The PCR mixture contained 2 μL of the 10-fold diluted cDNA template, 0.8 μL of the primer solution (containing 10 μM each of forward and reverse primers), 6.4 μL of Milli Q water, and 10 μL of SYBR Premix Ex Taq II (Takara Bio, Shiga, Japan). Transcript levels were normalized to the expression of *ACT4* measured in the same samples. The gene-specific primer pairs used are as follows: for *PR1b*, forward 5′-CTTGCGGTTCATAACGATGC-3′ and reverse 5′-TAGTTTTGTGCTCGGGATGC-3′; for *LOXd*, forward 5′-ATCTTGATGCTTTCACCGACA-3′ and reverse 5′-ACACTGCTTGGTTGCTTTTCTTC-3′; for *OPR3*, forward 5′-TCGTTTAATGAGGACTTTGAGGAAC-3′ and reverse 5′-AGGATTAGAGATGAAAAGACGACCA-3′; for *PI2*, forward 5′-ACGAAGAAACCGGCAGTGA-3′ and reverse 5′-TTGCCTCCACCGAAAACC-3′; for *Pti4*, forward 5′-TGTGATGGAGAAATGGCGAGTAGAT-3′ and reverse 5′-AGTTGATGGACACCTGTCAATTGTTC′; and for *ACT4*, forward 5′-TTGACTTGGCAGGACGTGA-3′ and reverse 5′-CAGCTGAGGTGGTGAACGAG-3′. The average of normalized transcript levels was compared between Met-treated and water-treated control plants (*n* = 8). The experiment was repeated three times.

### 4.5. Analysis of Defense Responses to Pathogen Infection

For time-course analyses of gene expression after *Pst* inoculation, two sets of 16 tomato plants of equal size were selected from 48 cultivated plants and treated with Met or water, respectively. Bacterial suspensions of *Pst* (1 × 10^5^ CFUs/mL) were prepared by a method similar to the pathogen inoculation assay. Bacterial suspensions were infiltrated into terminal and adjacent leaflets of the 3rd compound leaves of the Met-treated and water-treated control plants, followed by sampling of leaf tissues from the pathogen-infiltrated parts at 0, 8, 16 and 24 h after inoculation. At each time point, 8 RNA samples were prepared (2 samples from a single plant). The experiment was repeated three times.

For time-course analyses of gene expression after *Bc* inoculation, two sets of 12 tomato plants of equal size were selected from 48 cultivated plants and treated with Met or water, respectively. Spore suspensions of *Bc* (1 × 10^6^ spores/mL in 1.2% potato dextrose broth) were prepared by the same method as the pathogen inoculation assay. Spore suspensions were sprayed to all leaflets of excised 3rd compound leaves of the Met-treated and water-treated control plants, followed by sampling of leaf tissues from terminal and adjacent leaflets at 0, 12 and 24 h after inoculation. These were used for RT-PCR analysis. At each time point, 8 RNA samples (2 RNA samples from each plant) were prepared. The experiment was repeated three times.

### 4.6. Statistical Analysis

The data are presented as the means ± standard errors (SEs). Statistical analysis was performed using GraphPad Prism 9 software (GraphPad Software, Boston, MA, USA). Student’s *t*-test was used to analyze significant differences between Met-treated and water-treated groups. A *p*-value of less than 0.05 was considered statistically significant.

## Data Availability

Data are available on request.

## Supplementary Materials

The following supporting information can be downloaded at https://www.mdpi.com/article/10.3390/ijms25126315/s1.
