# Supplementary material for: Priming of Immune System in Tomato by Treatment with Low Concentration of L-Methionine
Source: Int J Mol Sci. 2024 Jun 7;25(12):6315. doi: 10.3390/ijms25126315 (PMC11204331; doi:10.3390/ijms25126315)
Supplement: Supplementary file 1 [file ijms-25-06315-s001.zip › ijms-2997966-supplementary.pdf]

A   B

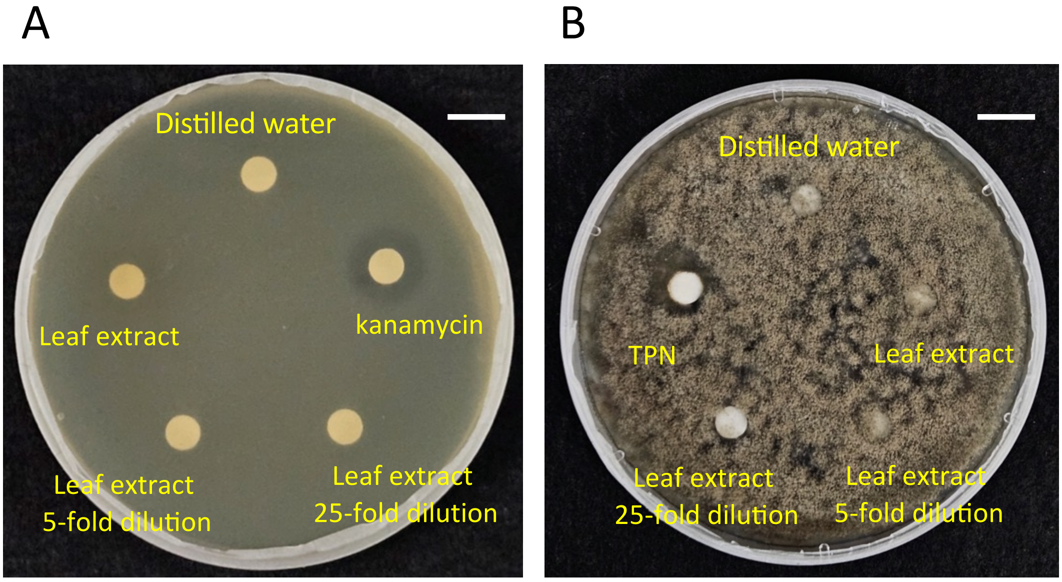

Figure S1. Antimicrobial activity of of leaf extract from L-methionine-treated tomato.

Leaf extracts were prepared by homogenization and centrifugation from leaf samples (100 mg) taken 2 days after treatment with L-methionine (Met). Paper disks (6mm, Whatman) containing 10 µl of leaf extract or its diluents were placed on culture plates. Scale bar, 10 mm.

(A) Antibacterial activity of leaf extract from Met-treated tomato against *Pseudomonas syringae* pv. *tomato* DC3000 (*Pst*). Nutrient broth medium plates containing *Pst* (1 x 10^5 CFU/ml) were used and cultured for 2 days at at 28℃. Kanamycin (50 µg/ml) and distilled water were used as positive and negative controls, respectively.

(B) Antifungal activity of leaf extract from Met-treated tomato against *Botrytis cinerea (Bc)*. Potato dextrose agar plates sprayed with *Bc* spore suspension (1 $\times$ 10^6 spores/mL in 1.2% potato dextrose broth) were used and cultured for 4 days at 20℃. TPN (2,4,5,6-tetrachloroisophthalonitrile) (20 mg/ml) and distilled water were used as positive and negative controls, respectively.
